# Editorial: Reward Processing in Motivational and Affective Disorders

**DOI:** 10.3389/fpsyg.2016.01288

**Published:** 2016-08-30

**Authors:** Frank Ryan, Nikolina Skandali

**Affiliations:** ^1^Centre for Mental Health, Division of Brain Sciences, Imperial College LondonLondon, UK; ^2^Department of Psychiatry, Behavioural and Clinical Neuroscience Institute, University of CambridgeCambridge, UK

**Keywords:** reward, anhedonia, depression, schizophrenia, addiction, computational neuroscience, Bayesian models

Reward prediction and valuation are central to decision making (Schultz et al., 1997; O'Doherty, 2004), and thus motivate and guide human action. Faulty reward processing can be pragmatically viewed as compromised decision-making, reflected in making suboptimal choices. A primary aim of this Research Topic is to provide a dimensional approach for reward processing. In accordance with the Research Domain Criteria, a newly proposed research classification of mental health disorders based on behavioral dimensions and neurobiological findings (Insel et al., 2010), reward processing deficits can be of transdiagnostic importance. This prominence has been largely influenced by the evolution of novel neuroimaging techniques, experimental cognitive psychology findings, and the application of computational modeling in simulating human behavior. This body of work has increased knowledge of the neural mechanisms underlying aberrant reward processing.

We maintain that construing patterns or expressions of reward processing as potential biomarkers, or indices of psychological vulnerability, can facilitate early detection and intervention in the clinical arena. Additionally, therapeutic approaches originating in the psychology laboratory aimed at modifying or reversing cognitive biases or behavioral approach biases linked to aberrant reward processes are showing promise in preventing relapse in the context of addiction (see Gladwin et al., 2016). It is this twin promise of enhanced prediction of vulnerability or risk and ultimately improved clinical outcomes, combined with a deeper understanding of brain functions, that motivated us to gather together this unique series of articles linked by the common thread of reward processing.

The *Topic* includes four original articles exploring reward processing in schizophrenia, depression, addiction and in the context of stress or anxiety. These empirical contributions are complemented by three review articles, two theoretical contributions and an opinion piece. In tandem with laboratory findings, these more conceptual articles put emphasis on the role of the integrity of neuromodulatory systems implicated in reward processing as well as the remarkable insights that can be derived from the implementation of computational modeling.

Rømer Thomsen, set the scene by outlining the subcomponents of reward processing: wanting, liking and learning. This parsing of reward processing enables a critical analysis of the concept of anhedonia, suggesting that deficits in reward processing are not restricted to “liking” (the subjective, pleasurable, experience of being rewarded). Other components, “wanting” and mechanisms underlying learning about rewards, can also be disrupted and contribute to the development and maintenance of disorders such as addiction and depression.

Arrondo et al. demonstrated blunted reward anticipation in people with a diagnosis of schizophrenia and depression. This attenuated striatal response to the prospect of monetary reward correlated with depressive symptoms in the schizophrenia group, but did not cohere with clinical symptoms of depression in the depressed cohort. Di Lemma et al. and colleagues found that, contrary to predictions, approach and avoidance tendencies following positive and negatively themed videos *did* change in parallel challenging the expectation that these are independent processes. Woud et al. investigated broadly similar processes investigating cohorts of current or former tobacco smokers. The researchers reported no attentional or behavioral approach biases in either current smokers, nicotine deprived smokers and ex-smokers, thus challenging incentive theories of addiction. These three innovative studies raise important questions for further research, and refine experimental methods in the process.

Robinson et al. applied a stress manipulation paradigm in order to study the effect of acute stress on two well-established biases in decision-making, temporal discounting and the framing effect. The researchers observed mood alterations in response to experimentally induced stress, but no effects on decision-making processes. Acute stress impacted on low level “bottom–up” perceptual biases, but higher level executive processes were unperturbed. The findings support the application of psychotherapeutic approaches aiming to enhance cognitive control as an apparently resilient component of therapeutic intervention for affective disorders.

Chekroud discussed the distortions in reward sensitivity and/or reward learning that contribute to the development of depression. He described the implementation of the free-energy principle, which views the brain as a “predictive machine,” aimed at reducing surprise (i.e., free energy) by constructing congruent cognitive models and optimizing actions. One potential clinical application is that changing cognitive representations using pharmacological agents or psychotherapy will be a necessarily gradual rather than immediate process. Also on the topic of depression, Dillon assigned a pivotal role to reward processing in the formation of long term memories. He concluded that impaired reward processing reflected distorted mesolimbic dopaminergic transmission, thus impeding the transfer of short-term memories into long-term episodic memory storage. Consequentially, in order to recover from depression, not only will somebody who is depressed need to overcome a memory bias for the recall of negative events, they will also struggle to recall positive events.

Cousijn highlighted the role of fronto-parietal and limbic brain networks that are implicated in vulnerability to cannabis use disorders, other substance misuse disorders and increased risk of anxiety and depression. When these cognitive control systems are compromised individuals are more likely to reach out for immediately available rewards whether they are linked to substance use or the powerful negative reinforcement that occurs when emotional distress is alleviated by avoidance, thus increasing the possibility of anhedonia and depression.

Moutoussis et al. differentiated between optimal decisions delivering the best possible rewards and a conceptualization of psychiatric disorder based on suboptimal reward processing. In this context, rewards are milestones or surrogates to strategic goals such as health, wellbeing and social affiliation. The researchers emphasized the importance of considering the patient's autonomy by pointing out the need for the clinician to engage in a dialog to elicit the patient's values and goals. Story et al. identified two factors involved in delayed reward discounting in psychiatric disorders: the opportunity cost that waiting for a delayed reward entails and the associated uncertainty of reward delivery.

The theoretical insights and experimental findings presented in this topic justify further exploration of the mechanisms underlying the anticipation, valuation and pursuit of rewards. In tandem with this, adapting and applying these findings in clinical settings could, we believe, provide additional therapeutic benefit.

## Author contributions

All authors listed, have made substantial, direct and intellectual contribution to the work, and approved it for publication.

## Funding

Dr. NS is supported by a UK Medical Research Council Doctoral grant studentship. Dr. FR wishes to acknowledge the support and encouragement he received from his employer Camden & Islington NHS Foundation Mental Health Trust and the Centre for Mental Health, Imperial College.

### Conflict of interest statement

The authors declare that the research was conducted in the absence of any commercial or financial relationships that could be construed as a potential conflict of interest.
